# A Comparative Study of Urban Park Preferences in China and The Netherlands

**DOI:** 10.3390/ijerph19084632

**Published:** 2022-04-12

**Authors:** Pauline van den Berg, Minou Weijs-Perrée, Gamze Dane, Esther van Vliet, Hui Liu, Siao Sun, Aloys Borgers

**Affiliations:** 1 Urban Systems and Real Estate Unit, Department of the Built Environment, Eindhoven University of Technology, 5612 AZ Eindhoven, The Netherlands; minouperree89@gmail.com (M.W.-P.); g.z.dane@tue.nl (G.D.); esthervanvliet96@gmail.com (E.v.V.); a.w.j.borgers@tue.nl (A.B.); 2 Key Laboratory of Regional Sustainable Development Modeling, Institute of Geographic Sciences and Natural Resources, Chinese Academy of Sciences, Beijing 100101, China; liltlh1999@163.com (H.L.); suns@igsnrr.ac.cn (S.S.); 3 College of Resources and Environment, University of Chinese Academy of Sciences, Beijing 100049, China

**Keywords:** parks, urban green, stated-choice, virtual environment, preferences, comparative study

## Abstract

Urban parks play an important role in tackling several urban challenges such as air pollution, urban heat, physical inactivity, social isolation, and stress. In order to fully seize the benefits of urban parks, it is important that they are attractive for various groups of residents. While several studies have investigated residents’ preferences for urban park attributes, most of them have focused on a single geographical context. This study aimed to investigate differences in park preferences, specifically between Dutch and Chinese park users. We collected data in the Netherlands and China using an online stated choice experiment with videos of virtual parks. The data were analyzed with a random parameter mixed logit model to identify differences in preferences for park attributes between Chinese and Dutch citizens, controlling for personal characteristics. Although the results showed a general preference for parks with many trees, several differences were found between the Dutch and Chinese respondents. These differences concerned vegetation (composition of trees and flowers), the presence of benches and play facilities, and could probably be explained by differences in park use, values of nature, and landscape preferences. The findings of this study can be used as design guidelines by urban planners and landscape designers to design attractive and inclusive parks for different target groups.

## 1. Introduction

Cities are currently dealing with several challenges. The urban population is growing as a result of an increasing world population and urbanization. Urban areas are often unhealthy places to live, characterized by heavy traffic, pollution, noise, social isolation, poor housing conditions, stress, and urban heat, resulting in the lower life expectancy of urban dwellers [1]. Urban green can play a role in tackling several of these challenges [2]. For example, urban parks can reduce heat, absorb noise, reduce air pollution, and store rainwater [3]. Additionally, living in an environment with more greenery positively influences well-being, as green helps people to relax and restore from stress [4,5], offers opportunities for social interaction [6], and promotes physical activity [7]. Moreover, it was found that spending more time in a park improves life satisfaction [8], and people who visited green spaces with a higher diversity of plants are happier [9]. Likewise, urban green spaces with greater biodiversity are likely to be associated with more positive emotional responses [10]. Thus, it is generally acknowledged that urban green is important for public health. In order to be able to design urban green spaces that are beneficial and attractive for different groups of urban residents, it is important to gain insights into the preferences of different target groups regarding urban park attributes. Based on these insights, guidelines can be derived for urban park designers and managers on what elements to include in a park.

Therefore, several studies have investigated urban park preferences. Research has shown that several spatial characteristics of parks influence people’s park preferences and experiences (e.g., [11,12,13]). First of all, both type and density of vegetation play a role in people’s preferences [14,15,16,17,18]. Size and accessibility [17,19] of green are also relevant, and the presence of facilities such as playground equipment and benches have also been found to be preferred [12,20]. Cleanliness and maintenance are also likely to play a role in the preferences of park users [17,21,22], although not all studies have found significant effects [18]. In a Chinese study [17], quietness and beautiful views were also important reasons for using green spaces. Finally, the presence of other people [11], presence of water [16,23], and noise [24] are important for park users.

Park preferences have been found to vary between different groups of people. Personal characteristics such as age [14,18,25], gender [18,26], household composition (presence of children in the household) [18], education level [15,18,27,28,29], and urban vs. rural place of residence [27] have been found to affect park preferences.

Moreover, several studies have focused on ethnicity as a determinant of park preferences. While people from various ethnic backgrounds have all been found to prefer natural environments over built environments [22], several studies have indicated that people from distinct ethnicities have different preferences for urban park attributes [13,30,31] For instance, Ho et al. [13] studied park preferences in the United States and found that Hispanics and African-Americans preferred more the recreational facilities and traditional park landscapes. The study of Kaplan and Talbot [32] indicated that African-Americans did not prefer dense vegetation. Gobster [21] found that people with an Asian background valued the park’s scenic beauty more, and people with a Latin American background the fresh air and lake effect. White people valued the trees and other park vegetation and African-Americans the facilities, maintenance, and activities. In a similar vein, Payne et al. [25] found that African-Americans tended to prefer the function of recreation over the conservation of park land.

Differences in park preferences between people from different geographical areas could be explained by the fact that they have varying ‘images of nature’ [31], or differences in landscape preferences [31] or landscape styles [33]. People may have a preference for vegetation and landscape types that they are more familiar with. Yu [27] compared park scene ratings from Chinese and Western groups and only found weak differences. However, they indicate that “for some specific Chinese landscapes, macro-cultural differences do occur because the ‘foreigners’ lack the knowledge of cultural meanings embodied in the landscapes” [27]. According to Yang and Kaplan [33], a Western landscape style is based on geometry and symmetry, while an Oriental landscape style is non-geometrical and asymmetrical. However, these landscape styles and values of nature seem to change over time. Traditionally, Chinese parks (or gardens) consisted of an enclosed landscape with a winding path to a quiet place [34]. However, a process of globalization and Westernization has resulted in an increased number of parks with large lawn areas in Chinese cities and a growing preference for neatly maintained landscapes, though often with limited public access [34] or with entrance fees [17]. Moreover, the study by Buijs et al. [31] indicated that rather than symmetrical parks, “Native Dutch people are strong supporters of the wilderness image, while immigrants generally support the functional image” [31].

Moreover, differences in park preferences between people from different countries could be explained by the fact that they use parks for different activities, and thus value parks for different reasons [22]. Özgüner [22] found that Turkish people in Turkey used parks for passive activities (resting, relaxing or picnicking), whereas Western people had a more active park use (walking, dog walking, or sports). Similarly, Yang et al. [34] indicated that Chinese people used parks for sitting and resting and social activities rather than active activities. The same differences between Chinese and Western residents were found in the United States [35]. Kloek et al. [36] studied participation and outdoor recreational behavior of Turkish and Chinese immigrants compared to non-immigrants in the Netherlands. Their findings showed that respondents of Chinese descent participated less often in recreational activities and mainly participated in individual-based activities such as walking, cycling, running, relaxing, yoga, and photography. According to Jim and Chen [17], the main purposes of the residents of Guangzhou for using green spaces are relaxation, quietude, physical exercise, nature appreciation, and aesthetic pleasure. Relaxation and enjoyment of nature as well as socialization and exercise are also mentioned by the residents of Singapore as being very important [37].

Although the role of ethnicity in park preferences has received some attention, the vast majority of studies into urban park preferences have focused on a single geographical context. Moreover, while some studies have focused on differences between native residents and immigrants in one country, only a few studies have compared park preferences of Western and non-Western groups living in different countries. This study aimed to contribute to this unexplored field by comparing the park preferences of Dutch residents in the Netherlands and Chinese residents living in China.

This study is an extension of the study of Van Vliet et al. [18] in which we explored the influence of urban park attributes on user preferences using an online stated choice experiment in the Netherlands. Results showed that participants particularly valued a high number of trees and flowerbeds with a diversity of flowers, and to a lesser extent, the presence of benches and play equipment. Two groups were identified in that study, namely a group that could be described as a “nature-loving group” and a group that could be described as an “amenity-appreciating group”. The study indicated that non-Dutch respondents were more likely to belong to the amenity-appreciating class, while the Dutch were more likely to specifically value the trees and flowers. However, the non-Dutch group was too small to draw any conclusions on the effects of ethnicity.

In order to assess to what extent the preferences of the Dutch are generalizable to other nationalities and geographical contexts, the aim of this study was to explicitly compare the preferences of two distinct samples, namely, a group of Dutch respondents and a group of Chinese respondents. These groups were selected because they differed significantly in geographical location, climate, and indigenous vegetation as well as in activities and values.

The current study extends the study of Van Vliet et al. [18] by using data of 540 Dutch respondents (respondents with a different ethnicity were removed from the sample), complemented with data that were collected from 719 Chinese respondents living in China using the same online survey as in the Dutch context. In this study, the pooled data of 1259 respondents were analyzed with a random parameter mixed logit model to identify differences in the preferences for park attributes between Chinese and Dutch respondents. The model controlled for the effects of personal characteristics (e.g., age, gender, work status and disability).

The rest of this paper is organized as follows. Section 2 describes the data and methods, followed by the results in Section 3. In Section 4, the findings are discussed and directions for future research are presented. A short conclusion completes the article.

## 2. Materials and Methods

While most studies on park preferences use a qualitative approach consisting of on-site interviews with park users (e.g., [21,22,24]), some have used a quantitative approach consisting of surveys asking respondents to rate the importance of several park attributes [13] or a conjoint method in which they let participants evaluate several park alternatives [11,12,14]. While the qualitative approach allows for in-depth investigation of a problem, the number of respondents for these studies is usually low. Quantitative approaches such as the conjoint analysis method allows for data to be gathered on preferences of large amounts of people. Therefore, in this study, to investigate the differences between Dutch and Chinese preferences for parks, a stated choice experiment was conducted. The same research design and method of data collection was used as described in [18].

### 2.1. Setup Stated Choice Experiment

**Figure 1:**
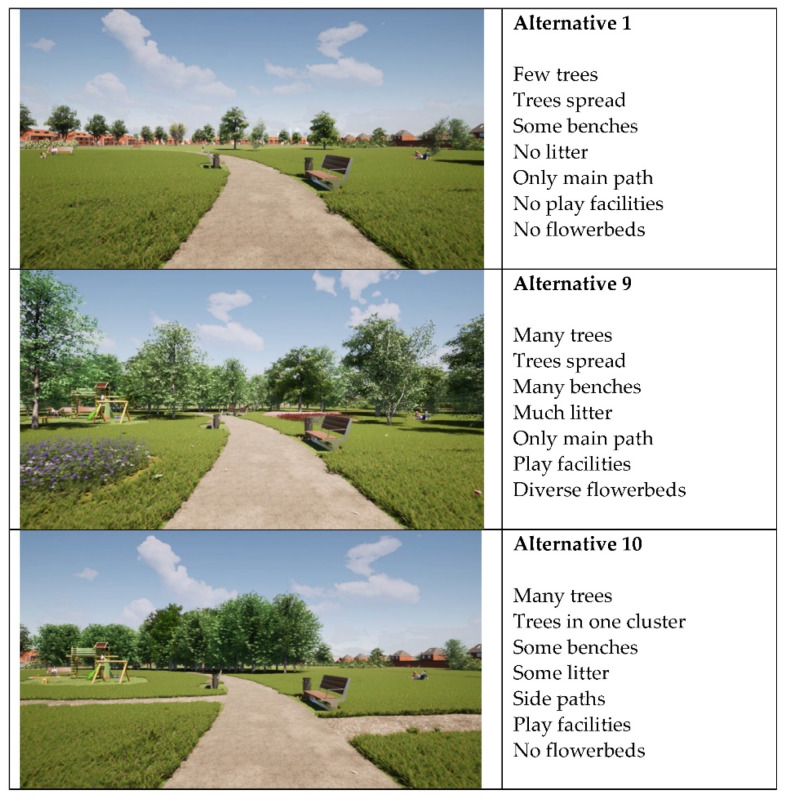
Screenshots of different park alternatives with varied attributes.

Based on a literature review and an expert meeting (see [18]), the attributes and attribute levels listed in Table 1 were selected. In order to create choice alternatives, these attribute levels were combined according to an orthogonal experimental design, generating 16 alternative parks. The hypothetical parks were presented using videos, whereby each video represented a walk through the park. Figure 1 shows screenshots of three alternatives with varying attributes.

**Figure 2:**
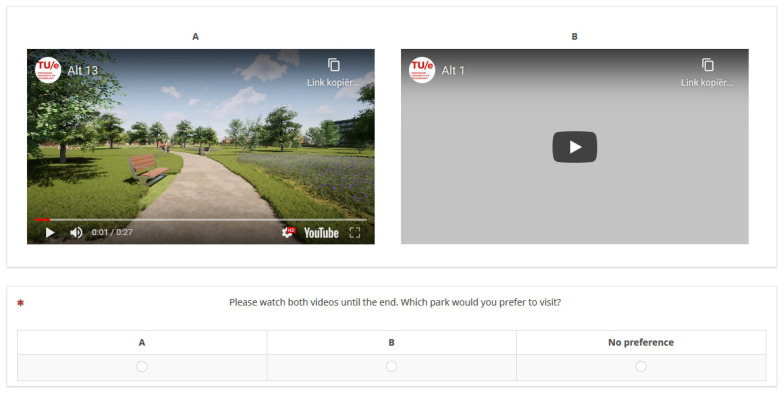
Screenshot of a choice task.

Choice sets were created by randomly combining two alternatives. Per choice set, the two videos were shown next to each other on the screen. Respondents were asked to watch both videos one after the other rather than simultaneously, so that they could pay attention to each video. They were asked to watch each video until the end and then answer the question “Which park would you prefer to visit?”. To each of these pairs, a ‘no preference’ option was added, resulting in three alternatives per choice set. The ‘no preference’ option allowed us to estimate a constant, which represents the likelihood that respondents choose one of the two videos as the preferred one. Figure 2 shows a screenshot of a choice task, where video A is playing (Video S1).

### 2.2. Data Collection

Data were collected by means of an online questionnaire. Watching the video of a hypothetical park took 26 s. As one choice task contained two videos, handling one choice task took roughly 1 min. To limit the total duration of the questionnaire, only four choice sets were presented to each respondent.

The Dutch respondents were recruited via the survey panels of two cities in the south of the Netherlands, namely, Hertogenbosch and Eindhoven and via social media (see [18]) for more information. The Chinese respondents were recruited via an online survey platform that is accessible to all of the public between 30 September and 18 November 2020 (www.wjx.cn).

The Dutch study was approved by the Ethical Review Board of the Built Environment Department of the Eindhoven University of Technology. Respondents had a chance of winning one of ten gift cards worth 25 euros. On completion of the online survey, the respondents in China received 0.1~10 RMB at random.

### 2.3. The Random Parameter Mixed Logit Model for Data Analysis

The random parameter mixed logit model was used to analyze the stated choices of all respondents. This is a more advanced version of the well-known multinomial logit model (see e.g., [38,39]), taking into account the panel structure of the data and taste heterogeneity. The basic multinomial logit (MNL) model is defined as:

(1) $$P_{i}=\frac{e^{V_{i}}}{\sum_{j}e^{V_{j}}}$$

where *P_i_* is the probability that an individual chooses alternative *i* from a set of alternatives, and *V_i_* is the structural utility of alternative *i*. The structural utility is the sum of weighted X-variables:

(2) $$V_{i}=\sum_{n}\beta_{n}X_{in}$$

The X-variables represent the levels of the attributes by means of dummy coding, resulting in L-1 parameters for each attribute with L levels (see Table 2). Per attribute, the expected least attractive level was coded 0 or 0 0, depending on the number of levels. Therefore, $X_{in}$ represents the score of the *n*-th variable (*n* = 0,…11; see Table 2) of alternative *i*. $\beta_{n}$ is a parameter to be estimated for variable *n*. In the basic MNL model, the *β*-parameters represent the mean weights of the variables. However, the random parameter model not only estimates the mean effect of the variables, but also determines the standard deviation around the mean. This can be denoted as:

(3) $$V_{i}=\sum_{n}\beta_{n}^{*}X_{in}$$

with $\beta_{n}^{*}$ being a parameter randomly drawn from a normal distribution with mean $\beta_{n}$ and standard deviation $\sigma_{n}$. The size of the standard deviation represents the amount of taste heterogeneity in the sample regarding that variable. The utility of the hypothetical alternatives depends on the attribute levels, represented by the X-variables (*n* = 1,…11). The utility of the ‘no preference’ option is defined by the constant (=1 for *n* = 0) and all other X-variables (*n*= 1, … 11) are equal to 0.

We wanted to measure the differences between the preferences of Dutch and Chinese respondents. This means that we should test for differences in the parameters between the two samples. Therefore, we used contrast parameters (see e.g., [40]). To estimate these contrast parameters, the specification of the utility of the alternatives has to be extended by adding a contrast variable. We added a *q*-index to differentiate between the two samples of respondents:

(4) $$V_{iq}=\sum_{n}\beta_{n}^{*}X_{in}+\delta_{n}\Delta_{q}X_{in}$$

The contribution of a variable was then measured as ($\beta_{n}^{*}$ + *δ_n_*∆*_q_*)*X_in_*, where $\beta_{n}^{*}$ is the random parameter for the *n*^th^ variable, contrast parameter *δ_n_* measures the difference between the mean $\beta_{n}$ for the Dutch and Chinese respondents regarding the *n*^th^ variable, and contrast variable ∆*_q_* is defined as +1 for Dutch respondents and −1 for Chinese respondents. If the *δ_n_*-parameter is significant, the mean weight of variable *n* differs significantly between the two samples. In addition, we estimated the standard deviation of the β-parameters for both countries separately, resulting in $\sigma_{nc}$, with c representing the Netherlands or China.

As the samples were quite different in some personal characteristics, (these personal characteristics should also be taken into account. Therefore, the interactions between personal characteristics and X-variables were added as follows:

(5) $$V_{iqc}=\sum_{n}(\beta_{n}^{*}X_{in}+\delta_{n}\Delta_{q}X_{in}+\sum_{j}\gamma_{njc}\Delta_{q}X_{in}Z_{qj}\;)$$

Therefore, for each *X*-variable *n*, the product of $\Delta_{q}X_{in}$ with each of the 10 (see Table 3) personal characteristics ($Z_{qj},\;j=1\ldots 10$) was added. The personal characteristics were effect coded (see Table 3) as the mean effect of effect coded variables is equal to 0. If $\gamma_{njc}$ is significant, the *j*-th personal characteristic influences the preferences. Note that the effects of the personal characteristics may differ per country *c* (*c* *ϵ* {NL, CN}). For each country separately, Equation (5) can be rewritten as:

(6) $$V_{iqc}=\sum_{n}\left(\beta_{n}^{*}X_{in}+[\delta_{n}X_{in}+\sum_{j}\gamma_{njc}X_{in}Z_{qj}]\right),\;c=\mathrm{NL}$$

(7) $$V_{iqc}=\sum_{n}\left(\beta_{n}^{*}X_{in}-\left[\delta_{n}X_{in}+\sum_{j}\gamma_{njc}X_{in}Z_{qj}\right]\right),\;c=\mathrm{CN}$$

The model was estimated using a stepwise approach. First, a multinomial logit was estimated, and insignificant interaction effects were removed from the model, starting with the most insignificant effects, until all remaining interaction effects were significant at a *p*-level of 0.15. Next, the random components were added (switching to the random parameter mixed logit model) and the interaction effects not significant at a *p*-level of 0.10 were further removed. This was conducted to ease the interpretation of the model. The mixed logit model was estimated by using 1000 Halton draws to calculate the simulated probabilities, and by taking into account the panel structure of the data (four observations per respondent).

## 3. Results

### 3.1. Sample Description

Table 4 shows the descriptive results of the sample characteristics. The average age of people in the sample from China was much lower (M = 38 years) compared to the average age of people from the sample from the Netherlands (M = 56 years). The Chinese sample consisted mainly of young respondents, while the majority of the Dutch sample belonged to the category of 55 and over. The total sample consisted of slightly more men than women. As can be seen, the sample from the Netherlands consisted of less people working full-time compared to the Chinese sample. The sample from China consisted of considerably more people who were living in a household with children compared to the Dutch sample.

Regarding education, the high category consisted of respondents with at least a bachelor’s degree. Respondents with a lower education level belonged to the ‘low education’ category. In both samples, the share of highly educated people was about 60%. For the Netherlands, the middle-income category represented respondents with a net yearly household income between 30 and 50 thousand euro, while for China, the middle-income category was defined between 100 and 300 thousand RMB per year. Compared to the Dutch sample, more Chinese respondents were in the low-income group, which was expected as the Chinese respondents were younger than those from the Netherlands. Finally, most people in the total sample (80%) did not have any disabilities.

### 3.2. Random Parameter Mixed Logit (ML) Model Results

Table 5 shows the results regarding the random parameter ML model as specified by Equation (5). The model performed well with McFadden’s rho^2^ = 0.226. Regarding the ‘constant’ ($X_{i0}$), the mean effect ($\beta_{0}$) was significant and negative (−1.358). This means that the utility of the ‘no preference’ option ($X_{i0}$ = 1) was negative, although it should be noted that $\beta_{0}$ is a random value and can incidentally also take positive values. As $X_{in}$ is equal to zero for the hypothetical alternatives, the parameter does not affect the utility of these alternatives. Therefore, based on $\beta_{0}$, the probability that the ‘no preference’ option will be chosen is in general smaller than the probability a hypothetical alternative will be chosen. However, there were differences between the Dutch and Chinese respondents as some of the interaction effect (γ’s) related to the constant were significant. For young Dutch respondents, the mean utility of the ‘no preference’ option was even more negative (−1.358 − 0.996 = −2.354), but for older or ‘highly educated’ Dutch respondents, the mean utility increased by 0.571 and 0.274, respectively, making the ‘no preference’ option less unlikely to be chosen. Additionally, for the middle-aged Dutch respondents, the net effect was positive (0.996 − 0.571 = 0.425). On the other hand, Dutch part-timers were more reluctant to choose the ‘no preference’ option. For Chinese respondents with high incomes, the utility of the ‘no preference’ option increased by 0.29 and decreased by the same amount for the medium income group. Finally, the standard deviations regarding the error component were significant for both countries, meaning that there were significant taste differences within the Dutch and the Chinese respondents. As the standard deviation for Dutch respondents was larger than for Chinese respondents, it can be concluded that the likelihood of selecting the ‘no preference’ option showed larger differences between the Dutch than the Chinese respondents (apart from the effects of the personal characteristics).

The results regarding the attribute level ‘some trees’ were rather straightforward. For Dutch respondents, the mean part worth utility of ‘some trees’ was equal to 0.449 + 0.214 = 0.663 and equal to 0.449 − 0.214 = 0.235 for the Chinese respondents. Note that the *δ*-parameter for ‘some trees’ was significant, indicating a significant difference between both samples. Interaction effects with personal characteristics were insignificant. This simply means that ‘some trees’ increased the utility of a hypothetical alternative by on average 0.663 or 0.235, depending on the country. Thus, Dutch respondents attached more value to ‘some trees’ than the Chinese respondents. Taste differences were reflected by different standard deviations.

**Figure 3:**
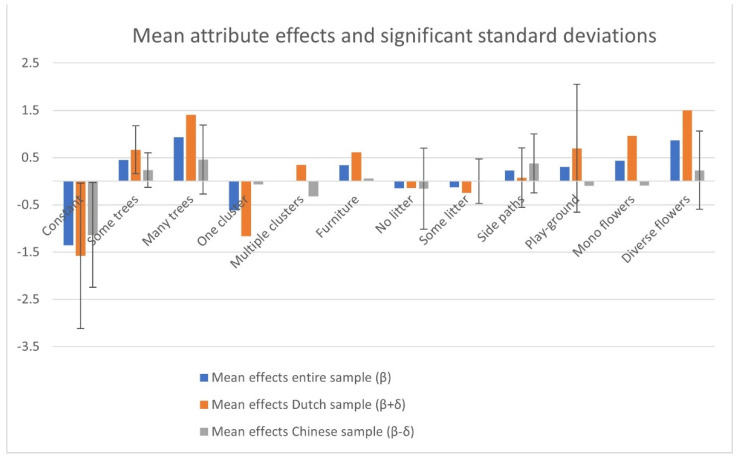
Mean attribute effects and significant standard deviations.

Now that the interpretation of the parameters has been explained, we concentrate on the main effects of the attributes (the β’s) and the main differences between the samples (the δ’s) regarding the attributes and the standard deviations (the σ’s). Note that the main reason for incorporating the interaction effects with personal characteristics was to reduce bias in these parameters. Figure 3 graphically presents the parameters.

Regarding trees, both samples in general preferred ‘some trees’ over ‘few trees’, and ‘many trees’ over ‘some trees’. Thus, the more trees, the better. For the Dutch respondents, this effect was clearly larger. Dutch respondents were not likely to prefer parks with just ‘one cluster of trees’, instead, they preferred ‘multiple clusters’ and to a lesser extent ‘trees being spread’ over the park. This was different for Chinese respondents, who preferred both ‘one cluster’ and ‘spread trees’ over ‘multiple clusters’. Just like with the number of trees, the Dutch respondents were more pronounced in their preferences.

Regarding furniture, the Dutch clearly preferred ‘many benches’ over ‘some benches’. The Chinese respondents did not have clear preferences regarding the number of benches. Remarkably, litter appeared to have no significant effect on the preferences. There were on average no differences between ‘no litter’, ‘some litter’, or ‘much litter’ for both samples. The Chinese respondents did show significant individual differences regarding their preferences for litter. Part of the Chinese respondents preferred ‘no litter’ or ‘some litter’ over ‘much litter’.

‘Side paths’ were slightly preferred over just one ‘main path’ by the Dutch sample, while the Chinese respondents clearly preferred the ‘side paths’. Now, both samples showed high standard deviations, indicating severe differences in preferences within each sample. The Chinese sample was on average not in favor of a ‘playground’, while the Dutch preferred having a ‘playground’ in the park, especially when they had children. Still, there was a lot of taste difference in the Dutch sample. Furthermore, regarding flowerbeds, the Chinese respondents did not have clear preferences on average, although the appreciation of ‘diverse flowerbeds’ differed considerable between the Chinese respondents. The Dutch respondents agreed that ‘monotonous flowerbeds’ and to a higher extent ‘diverse flowerbeds’ added value to parks compared to ‘no flowerbeds’ at all.

## 4. Discussion and Future Research Directions

This study aimed to gain more insights into the park preferences of Dutch and Chinese residents, especially the differences between these two groups. First, the negative constant indicates that respondents from both countries were very unlikely to choose the ‘no preference’ option. This suggests that they noticed differences between the alternative videos and had a preference of one alternative over the other. Next, the findings indicated that both groups preferred parks with ‘many trees’. Dutch respondents had a more outspoken preference for this attribute compared to the Chinese respondents. Dutch respondents preferred trees in multiple clusters or trees being spread over the park, while Chinese respondents preferred trees spread or trees in one cluster over multiple clusters. The Dutch also showed a stronger preference for flowerbeds, especially for ‘diverse flowerbeds’, compared to the Chinese respondents. This is in line with the study by Buijs et al. [31], who found that Dutch people tended to prefer wilderness images compared to immigrants in the Netherlands. In addition, a study by Gobster [21] showed that White people preferred trees and other vegetation, and Asian people valued the scenic beauty of a park more. Other studies [17,34] have indicated that Chinese residents increasingly valued parks with well-designed and maintained large green sites. Therefore, it is likely that Chinese people prefer more open parks compared to Dutch people, who prefer more trees and wilderness aspects.

The results showed a general preference for parks with ‘side paths’, although this preference was stronger among the Chinese respondents. This may not support the findings by Kloek et al. [36], who found that Chinese immigrants were more involved in individual activities such as walking and running, however, this study compared respondents from different countries. The high standard deviations indicated significant individual differences in preferences within each sample.

Dutch respondents were found to show a strong preference for parks with ‘many benches’, whereas Chinese respondents did not have a clear preference for benches. This is in contrast to [34], concluding that Chinese people used parks for sitting and resting. Dutch respondents were also found to prefer having a ‘playground’ in the park, especially when they had children, while the Chinese sample seemed indifferent regarding the playground. This might be related to the fact that Chinese people value parks for their quietness and beautiful views [17] and use them for less active activities [34].

The amount of litter was not found to affect the park preferences of either the Dutch or the Chinese, although the Chinese respondents showed significant individual differences regarding their preferences. This is in contrast to the findings of other studies that indicated that cleanliness and maintenance affected the park users’ preferences [11,21,22] and were specifically valued by Chinese residents [17,34]. A possible explanation for the fact that we did not find a significant effect of litter could be that the litter was not very notable in the virtual environments and the virtual environments generally looked rather clean. Moreover, the virtual environments did not include smells related to litter or dog excrement in real parks.

Table 6 shows the most preferred attribute levels for each country. As can be seen, there were clear differences between the two samples regarding the composition of trees and the presence of a playground. The Dutch respondents preferred multiple clusters of trees, while the Chinese respondents preferred trees to be spread or in one cluster. In addition, the Dutch sample preferred a playground, while the Chinese sample did not. While the Dutch had a preference for many benches, the Chinese had no clear preference regarding public furniture. Table 6 also shows that for cleanliness, the respondents of both countries had no clear preference. The overview in Table 6 can be used as guidelines by urban park designers.

While we found some significant differences in urban park preferences between the Dutch and Chinese respondents, it is not clear how these differences can be explained. As indicated in the introduction, several possible explanations exist. The differences in preferences could be due to differences in the preferred activity types at parks, or to differences in vegetation types or landscapes that people are familiar with. Further research is needed to investigate the mechanisms underlying these differences in preferences for park attributes.

Aside from differences between the two countries, several differences were found within the samples of Dutch and Chinese respondents related to personal and household characteristics such as age, gender, work status, income, household composition, and physical ability. This is in line with several other studies that found personal characteristics affected park preferences (e.g., [14,15,25,26,27,28,29]).

The significant standard deviations showed that there were preference variations related to the number of trees, litter, paths, playground, and flowerbeds. For urban designers, it is therefore important to take these differences into account and refrain from designing parks for ‘average’ residents. Parks should be inclusive and attractive to different target groups, varying in ethnicity, age, gender, and physical ability.

While this study has provided relevant insights in the park preferences of Dutch and Chinese residents, several directions for further research can be given. First, using a stated-choice approach limits the number of attributes that can be included. For instance, this study only manipulated the number and composition of trees, while other studies have found that the height of trees (as shelter, shade or to reduce (vehicular) noise) is important to predict people’s subjective well-being [41]. Future research could analyze the preferences for trees in more depth. In addition, preferences regarding types of flowers and wildlife habitats could be analyzed in more depth.

Moreover, we used only one specific park design as a baseline for the choice alternatives. As a result, the generalizability of the findings to other types of parks (with different sizes, types of vegetation, and amenities) is limited. The base park was designed by taking a typical Dutch neighborhood park into account, of around 3.5 hectares with grass, beeches and birches surrounded by semi-detached and detached houses, and three apartment blocks. It could also have been designed by taking into account a typical park in China.

Although the use of videos of virtual environments is useful and more reliable than static images to investigate preferences regarding environments [42], evaluations of virtual and real environments have been found to differ [43]. Moreover, the method of using videos of virtual environments is rather passive. Respondents might feel more engaged or more present in the environment when they can walk through and interact with the environment by using their keyboard or in an immersive VR environment.

Another limitation of this study concerns the maintenance of the park. While we manipulated the amount of litter, no significant preferences were found regarding this attribute. This might be due to the fact that the variations in cleanliness were more subtle than variations in other attributes. While the litter was of a realistic size, people may not have noticed the manipulated variable. Still, it would be expected that the degree of cleanliness in a park would be important to users, for instance, for their sense of safety. It would also be relevant to test the effect of smell in this regard. This could be included in a immersive virtual reality lab-based experiment with virtual parks, or in a study with real-time park environments.

Further research could focus on the design and presence of equipment and amenities such as litter bins, public toilets, dog walking areas, and dog toilets. In addition, it would be interesting to further examine the influence of the maintenance of greenery. Attributes such as the length of the grass, the presence of weeds, and the wilderness of the flowerbeds could also be manipulated in virtual parks. This is likely to make these virtual parks more realistic.

In our stated choice experiment, we only asked respondents to indicate which park alternative they preferred. However, we do not know why they preferred a park. Further research should aim to understand how urban park attributes affect satisfaction as well as affective experiences or emotions. This could help to design parks where people feel safe and happy, or experience a sense of place, which in turn can contribute to their subjective well-being.

Aside from the spatial attributes of the park, other aspects of a park visit such as type of activity, time of the day, company, and time spent could be important influences on people’s preferences and subjective well-being [44]. For example, people who visit a park for a walk on their own probably have different preferences than people who go during the afternoon to the park with their children. Future research on park preferences should incorporate these aspects.

Finally, this research could be expanded to other geographical or cultural contexts to further investigate differences in urban park preferences. In addition, more detailed research related to the use of parks and the effect on subjective well-being would be welcome. This could provide relevant guidelines for the design of inclusive parks that are attractive to different target groups.

## 5. Conclusions

This study used an online stated-choice experiment with videos of simulated parks to compare the preferences of Dutch and Chinese residents regarding different park attributes (number of trees, composition of trees, furniture, litter, side paths, a playground, and flowerbeds). Data of 1259 respondents were collected: 540 Dutch respondents and 719 Chinese respondents. The data were analyzed with a random parameter mixed logit model to identify differences in preferences for park attributes between the Chinese and Dutch, while controlling for the effects of personal characteristics. The results showed that the Dutch had stronger preferences for more trees and flowers, more benches, and play facilities, while the Chinese valued multiple paths in the park. There was a striking difference regarding the composition of trees. The Dutch liked parks with multiple clusters of trees and strongly disliked parks with only one cluster of trees. In contrast, the Chinese disliked parks with multiple clusters of trees. This study confirms that differences in park preferences exist between Dutch and Chinese residents. These differences are likely to be related to differences in park use (active vs. passive activities, individual vs. joint activities), different images of nature, or landscape preferences (e.g., a preference for wilder nature among the Dutch [31]). In addition to differences between the respondents of the two countries, the results showed significant standard deviations, indicating that there were taste differences in park preferences within the two samples. Personal characteristics were added to the model as control variables (to reduce bias) because the samples differed in these characteristics. While our aim was not to explicitly study the effect of personal characteristics on the preferences for park attributes, the significant interaction effects show that park preferences are related to age, household composition, income, and physical ability. The findings of this study can be used as design guidelines by urban planners and landscape designers to design attractive and inclusive parks for different target groups.

## Figures and Tables

**Table 1:** Selected attributes and their levels.

Attribute		Levels
1	Number of trees	Few trees
		Some trees
		Many trees
2	Composition of trees	Spread
		One cluster
		Multiple clusters
3	Public furniture	Some benches
		Many benches
4	Cleanliness	No litter
		Some litter
		Much litter
5	Paths	One main path
		One main path and multiple smaller paths
6	Playgrounds	None
		One playground
7	Flowers	None
		Three monotonous (i.e., single type) flowerbeds
		Three diverse flowerbeds

**Table 2:** Coding of attribute levels.

Attributes	Attribute Level	Coding
Constant	Hypothetical park preference	*X*_0_ = 0
	No preference	*X*_0_ = 1
Number of trees	Some trees	*X*_1_ = 1, *X*_2_ = 0
	Many trees	*X*_1_ = 0, *X*_2_ = 1
	Few trees (reference)	*X*_1_ = 0, *X*_2_ = 0
Composition of trees	One cluster	*X*_3_ = 1, *X*_4_ = 0
	Multiple clusters	*X*_3_ = 0, *X*_4_ = 1
	Spread (reference)	*X*_3_ = 0, *X*_4_ = 0
Public furniture	Many benches	*X*_5_ = 1
	Some benches (reference)	*X*_5_ = 0
Cleanliness	No litter	*X*_6_ = 1, *X*_7_ = 0
	Some litter	*X*_6_ = 0, *X*_7_ = 1
	Much litter (reference)	*X*_6_ = 0, *X*_7_ = 0
Paths	Side paths	*X*_8_ = 1
	One main path (reference)	*X*_8_ = 0
Playgrounds	Playground	*X*_9_ = 1
	None (reference)	*X*_9_ = 0
Flowers	Mono- flowerbeds	*X*_10_ = 1, *X*_11_ = 0
	Diverse flowerbeds	*X*_10_ = 0, *X*_11_ = 1
	No flowerbeds (reference)	*X*_10_ = 0, *X*_11_ = 0

**Table 3:** Coding of personal characteristics.

Personal Characteristic	Level	Coding
Gender	Female	*Z*_1_ = 1
	Male	*Z*_1_ = −1
	Other/Missing	*Z*_1_ = 0
Age	Younger than 35	*Z*_2_ = 1, *Z*_3_ = 0
	35–54	*Z*_2_ = −1, *Z*_3_ = −1
	55 and older	*Z*_2_ = 0, *Z*_3_ = 1
Occupation	Fulltime	*Z*_4_ = 1, *Z*_5_ = 0
	Parttime	*Z*_4_ = 0, *Z*_5_ = 1
	Unemployed/retired	*Z*_4_ = −1, *Z*_5_ = −1
	Missing	*Z*_4_ = 0, *Z*_5_ = 0
Education level	Low education	*Z*_6_ = −1
	High education	*Z*_6_ = 1
	Missing	*Z*_6_ = 0
Income level	Low	*Z*_7_ = 1, *Z*_8_ = 0
	Medium	*Z*_7_ = −1, *Z*_8_ = −1
	High	*Z*_7_ = 0, *Z*_8_ = 1
	Prefer not to answer	*Z*_7_ = 0, *Z*_8_ = 0
	Missing	*Z*_7_ = 0, *Z*_8_ = 0
Household	With children	*Z*_9_ = 1
	Without children	*Z*_9_ = −1
	Missing	*Z*_9_ = 0
Disability	Not disabled	*Z*_10_ = −1
	Disabled	*Z*_10_ = 1

**Table 4:** Sample characteristics of the respondents.

	The Netherlands (*n* = 540)		China (*n* = 719)		Total (*n* = 1259)	
	Mean	SD	Mean	SD	Mean	SD
Age	55.6	17.8	37.8	14.0	45.4	17.8
	*N*	%	*N*	%	*N*	%
Gender						
Female	247	46	310	43	557	44
Male	290	54	409	57	699	56
Other/Missing	3	1			3	
Age						
Younger than 35	91	17	356	50	447	36
35–54	120	22	261	36	381	30
55 and older	329	61	102	14	431	34
Occupation						
Fulltime	149	28	384	53	533	42
Parttime	131	24	103	14	234	19
Unemployed/retired	224	41	229	32	453	36
Missing	36	7	3		39	3
Education level						
Low education	182	34	298	41	480	38
High education	330	61	415	58	745	59
Missing	28	5	6	1	34	3
Income level						
Low	145	27	303	43	448	36
Medium	146	27	278	39	424	34
High	126	23	107	15	233	19
Prefer not to answer	121	22	31	4	152	12
Missing	2				2	
Household						
With children	104	19	442	62	546	43
Without children	421	80	266	37	687	55
Missing	15	3	11	1	26	2
Disability						
Not disabled	433	80	579	81	1012	80
Disabled	107	20	140	19	247	20

**Table 5:** Results of the random parameter mixed multinomial logit model.

Attribute Level (*X*’s)	Main Effects (*β*’s)	Differences (*δ*’s)	Standard Deviations (σ’s)		Interaction Effects (*γ*’s)								
					Young	Old	Female	Educ High	Part Time	Full Time	Income High	Child	Disabled
Constant	−1.358 ***	−0.221	1.5375 ***	NL	−0.996 ***	0.571 **		0.274 *	−0.562 ***				
			1.1084 ***	CN							−0.290 **		
Some trees	0.449 ***	0.214 ***	0.5058 *	NL									
			0.3671 *	CN									
Many trees	0.932 ***	0.474 ***	0.4536	NL	−0.733 ***	0.667 ***	−0.194 *				0.327 **		
			0.7257 ***	CN	0.243 ***				0.237 **				
One cluster	−0.616 ***	−0.550 ***	0.0042	NL	0.347 **	−0.478 ***					−0.222 *		0.213 **
			0.0259	CN	−0.133 **					−0.144 *			
Multiple clusters	0.015	0.331 ***	0.00055	NL					0.550 ***				
			0.0055	CN						−0.292 ***			0.253 **
Furniture	0.336 ***	0.275 ***	0.0028	NL									
			0.3576	CN									
No litter	−0.152	0.0068	0.3031	NL		−0.266 **							−0.419 ***
			0.8600 ***	CN							0.312 **		
Some litter	−0.124	−0.122	0.0252	NL			0.232 **						−0.346 **
			0.4678 **	CN		0.190 *							
Side paths	0.225 ***	−0.150 **	0.6278 ***	NL									
			0.6272 ***	CN	0.325 ***	−0.339 ***							
Play-ground	0.299 ***	0.399 ***	1.3511 ***	NL		0.330 **						0.336 **	
			0.3351	CN									0.200 **
Mono flowers	0.434 ***	0.527 ***	0.1566	NL						0.223 *			
			0.0803	CN									
Diverse flowers	0.866 ***	0.635 ***	0.1669	NL			0.210 **		−0.604 ***	0.417 **			
			0.8290 ***	CN	0.216 *	−0.309 **	0.150 **						

(***) Significant at 1% level. (**) Significant at 5% level. (*) Significant at 10% level.

**Table 6:** Most preferred attribute levels per country.

Attribute	Preference NL	Preference CN
Number of trees	Many trees	Many trees
Composition of trees	Multiple clusters	One cluster or spread
Public furniture	Many benches	No preference
Cleanliness	No preference	No preference
Paths	Side paths	Side paths
Playground	Playground	No playground
Flowers	Diverse flowerbeds	Diverse flowerbeds

## Data Availability

Data are available upon request to the corresponding author.

## Supplementary Materials

The following are available online at https://www.mdpi.com/article/10.3390/ijerph19084632/s1, Video S1: Video of alternative 7.
